# Lung function and the risk of frailty in the European population: a mendelian randomization study

**DOI:** 10.1186/s40001-024-01685-y

**Published:** 2024-02-01

**Authors:** Rong Zhou, Ge Tian, Xingzhi Guo, Rui Li

**Affiliations:** 1 Department of Geriatric Neurology, Shaanxi Provincial People’s Hospital, Xi’an, 710068 Shaanxi China; 2 Department of Geriatric Neurology, The Third Affiliated Hospital of Xi’an Jiaotong University, Xi’an, 710061 Shaanxi China

**Keywords:** Frailty index, Lung function, Mendelian randomization, Genome-wide association study

## Abstract

**Background:**

Epidemiological evidence has suggested a relationship between lung function and frailty, but the precise nature of the causality remains unclear. In this study, we applied a two-sample Mendelian randomization (MR) analysis to determine the causal effects of lung function on frailty.

**Methods:**

Single nucleotide polymorphisms (SNPs) independently related (*P* ≤ 5E−08) to lung function, as identified by genome-wide association study (GWAS), were applied as instrumental variables (IV). The association with frailty index (FI) was investigated using summary-level data from the latest GWAS on FI (*n* = 175,226). Different statistical methods were employed to evaluate the causal estimates between lung function and FI. The pleiotropy, heterogeneity, and leave-one-out analysis were applied to confirm the stability of the MR estimates.

**Results:**

Using the random-effect inverse-variance weighted approach, genetically proxied forced expiratory volume in the first second (FEV1), ratio of FEV1 on forced vital capacity (FVC) [FEV1/FVC], and peak expiratory flow (PEF) were significantly and inversely associated with FI (FEV1, *β* = −0.08, *P* = 2.03E−05; FEV1/FVC, *β* = −0.06, *P* = 9.51E−06; PEF, *β* = −0.07, *P* = 4.09E−04) with good statistical power (99.7–100%). However, no significant association was observed between FVC and FI (*β* = −0.01, *P* = 0.681). Leave-one-out analysis showed that there was no single SNP driving the bias of the estimates. There was potential heterogeneity, but no obvious pleiotropy was founded in this MR study.

**Conclusions:**

Our findings indicate that impaired pulmonary function is closely related to the risk of frailty. Enhancing lung function in the elderly population may contribute to the prevention of frailty to a certain extent.

**Supplementary Information:**

The online version contains supplementary material available at 10.1186/s40001-024-01685-y.

## Introduction

Aging poses a significant healthcare challenge for the elderly population, resulting in a huge economic burden worldwide. Among the various geriatric syndromes, frailty has emerged as an important field of medical research. Frailty is a multidimensional and potentially preventable clinical syndrome characterized by a decline in the body's physiological reserves and resilience [1]. A meta-analysis encompassing 467,779 hospital inpatients within the geriatric demographic disclosed a frailty prevalence rate of 47.4% [2]. Previously studies showed that frailty was associated with multiple adverse health outcomes, including dementia, falls, and increased hospital admissions [1, 3]. Thus, it is imperative to identify risk factors for frailty to implement timely interventions and avert adverse clinical events linked to frailty, improving the prognosis for the elderly.

Increasing evidence suggests that patients with chronic pulmonary disorders have an increased susceptibility to frailty [4, 5]. For instance, previous studies showed that frail individuals often encountered a decline in lung function earlier in comparison to their healthy controls in the community [4, 6]. In addition, a prospective cohort study demonstrated that the prevalence of frailty was obviously increased (10.2%) in patients with chronic obstructive pulmonary disease (COPD) [7]. Forced expiratory volume in the first second (FEV1), forced vital capacity (FVC), and peak expiratory flow (PEF) are the three common indicators used to evaluate lung function [8–10], which provide critical perspectives on the efficiency and capacity of the respiratory system. A recent longitudinal observational cohort study with 1188 participants indicated that higher FEV1 and FVC were associated with a lower prevalence (odds ratio [OR] ≈ 0.43) and incidence (OR ≈ 0.40) of frailty among the elderly in community [11]. According to a comprehensive meta-analysis that included 20 studies and 11,620 participants, the prevalence of frailty was estimated to be 32.07%, and patients with frailty had lower FEV1 than COPD patients [12]. Meanwhile, a study with 2559 community participants has showed a significant negative correlation between frailty and peak PEF [13]. However, the results of a cohort study in nursing home residents showed that PEF was not an independent factor associated with the incidence of frailty and falls after adjustment the potential confounding variables [14]. Since observational studies are susceptible to the influence of confounding factors and reverse causation, the existence of a causal link between lung function and frailty remains uncertain.

Mendelian randomization (MR) study has currently been widely applied to infer the etiology in epidemiology. Based on the principle of random, independent allocation of alleles during the parental inheritance, it is possible for offspring to generate random genetic variation. In MR analysis, this inheritance variation is usually treated as the instrumental variable (IV) for exposure [15]. As genetic variants are distributed in a manner similar to the random assignment of individuals in randomized controlled trials [16], MR supports an intuitive deduction of causality between potential risk factors and diseases, while diminishing the influence of confounding factors. Thus, we conducted a two-sample MR study to assess whether there is a causal relationship between lung function and frailty.

## Materials and methods

### Study design and instruments selection

**Fig. 1:**
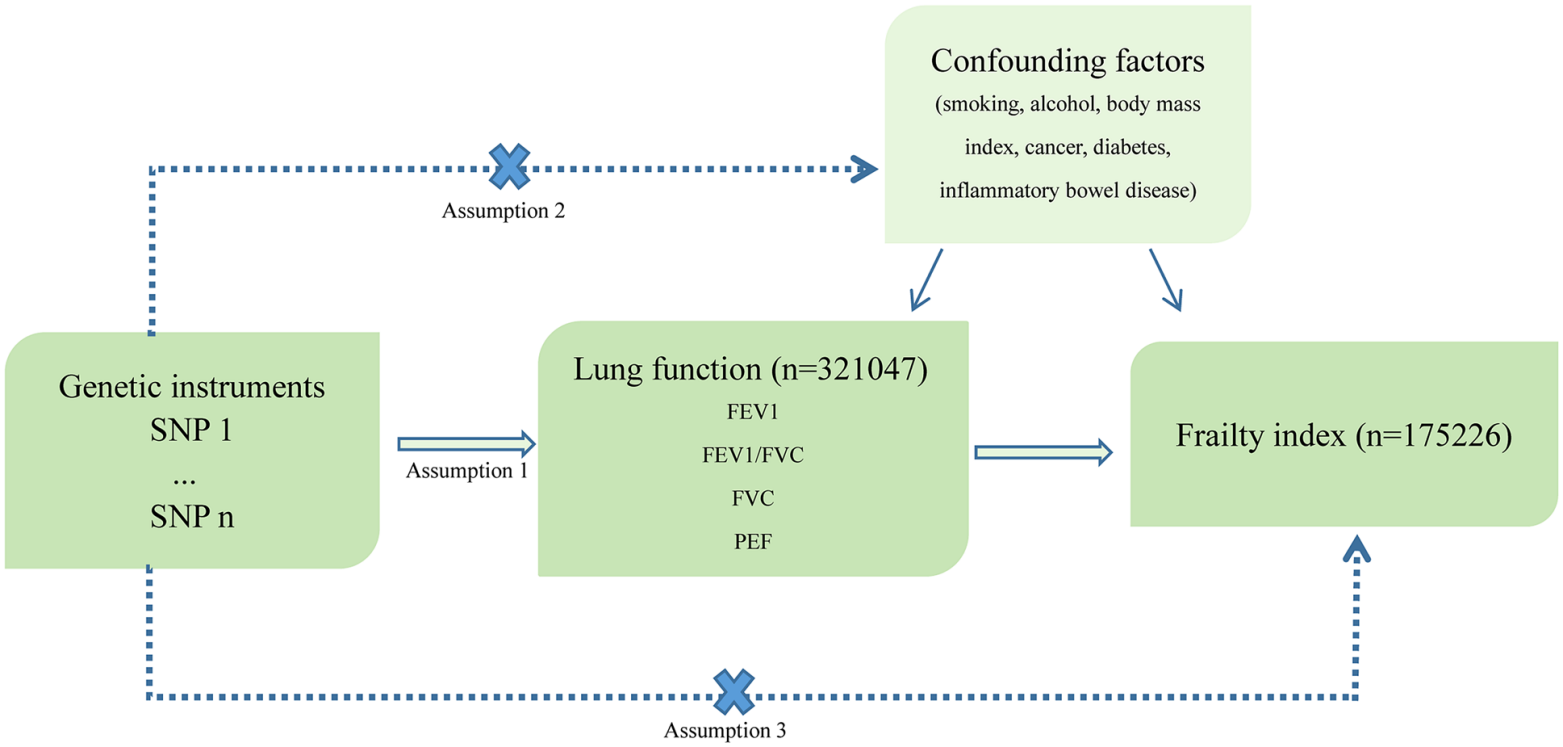
Study design for this Mendelian randomization study. Assumption 1: single nucleotide polymorphism (SNP) strongly associated with lung function; Assumption: SNP not related with confounding factors affecting both lung function and frailty; Assumption 3: SNP not associated with frailty. FEV1, forced expiratory volume in the first second; FVC, forced vital capacity; PEF, peak expiratory flow

Three assumptions need to be satisfied for a valid MR analysis (Fig. 1) [17]. Assumption 1 (Relevance): IV is significantly associated with lung function at the genome-wide level. Assumption 2 (Independence): IV is not correlated with potential confounding factors that may affect lung function and frailty. Assumption 3 (Exclusion restriction): IV does not directly lead to frailty, it can only affect frailty via lung function. Independent single nucleotide polymorphisms (SNPs) closely related to lung function (*P* ≤ 5E−08) were selected as IV from the summary statistics of genome-wide association studies (GWAS) on lung function [18]. Functional enrichment and pathway analyses showed that genes associated with these selected SNPs were primarily implicated in the organization of elastic fiber and extracellular matrix, as well as the ciliogenesis processes, which was significantly enriched in smooth muscle and the lung [18]. In addition, to eliminate the potential influence of confounding factors on both lung function and frailty (Fig. 1), we removed SNPs associated with smoking, alcohol consumption, body weight, body mass index, cancer, diabetes, coronary artery disease, inflammatory bowel disease, and irritable bowel syndrome through comprehensively retrieving the PhenoScanner V2 (http://www.phenoscanner.medschl.cam.ac.uk/) [19]. To adhere to the fundamental principles of MR analysis, we first calculated the *R*^2^ and *F*-statistic to evaluate the strength of the selected IV [20]. An *F*-statistic greater than 10 indicates a strong IV, enabling the exclusion of weak IV biases. Subsequently, those IV with linkage disequilibrium (LD, *r*^2^ < 0.001 within 10,000 kb) was clumped [21]. Finally, SNPs potentially associated with the corresponding outcome were also removed from the IV.

## Data sources

The GWAS summary statistics for lung function used in this study was meta-analyzed by *Shrine N *et al., which was based on the data from the UK Biobank and SpiroMeta Federation. The primary measures analyzed included FVC, FEV1, FEV1/FVC ratio, and PEF [18]. The GWAS data for FVC, FEV1, PEF, and FEV1/FVC ratio comprised 321,047 individuals of European descent aged 39 to 72, with 55.6% females. The details of IV estimation related to above lung function parameters are shown in Table 1. Frailty index (FI) is widely acknowledged as a measure for evaluating frailty. The FI refers to the deficit accumulation of multiple items such as symptoms, signs, laboratory abnormalities, and disease diagnosis (Additional file 2: Table S1), which is derived by calculating the proportion of observed deficits relative to the total number of considered deficits [22]. The details of the deficit items used for calculating the FI are available in Additional file 3: Table S2. Summary-level data on FI were extracted from the GWAS meta-analysis conducted by Atkins et al. which encompassed 175,226 participants of European descent [23]. Of these participants, 164,610 from the UK Biobank were aged 60–70 years (mean 64.1) with 51.3% females, and the other 10,616, from the TwinGene were aged 41–87 years (mean 58.3) with 52.5% females (Additional file 4: Table S3).

**Table 1:** The GWAS summary data on lung function and frailty used in this study

Exposure/outcome	Author	Year	Sample size (*N*)	SNPs	PMID
FEV1	Shrine N et al.	2019	321,047	19,674,931	30,804,560
FEV1/FVC	Shrine N et al.	2019	321,047	19,671,887	30,804,560
FVC	Shrine N et al.	2019	321,047	19,676,344	30,804,560
PEF	Shrine N et al.	2019	321,047	14,791,652	30,804,560
Frailty index	Atkins JL et al.	2021	175,226	7,589,717	34,431,594

*GWAS* genome-wide association studies, *FEV1* forced expiratory volume in the first second, *FVC* forced vital capacity, *PEF* peak expiratory flow, *N* number, *SNP* single nucleotide polymorphism

## Mendelian randomization analysis

The causal relationship between lung function and frailty was assessed using the random-effect inverse variance weighted (IVW) method, which is widely applied in MR analysis. In addition, we also employed MR-Egger and weighted median methods as sensitivity analyses to validate the stability of the estimates. The MR Pleiotropy RESidual Sum and Outlier (MR-PRESSO) test was further used to identify outliers and exclude any peripheral SNPs that might influence the results, and the MR-Egger intercept test was conducted to assess the horizontal pleiotropy [21, 24]. Additionally, to ensure the accuracy and credibility of MR analysis results, we conducted the Cochran's *Q*-statistic test to assess the heterogeneity of the IV. Moreover, to evaluate the impact of each SNPs on overall MR outcome, leave-one-out sensitivity test was implemented via conducting permutation analysis by eliminating one IV each time. Statistical power for the MR analysis was calculated as previously described [25]. The statistical analysis was conducted using the TwoSampleMR package in the R software, a widely used tool for MR studies [26]. A *P*-value less than 0.05 was deemed statistically significant.

## Results

**Fig. 2:**
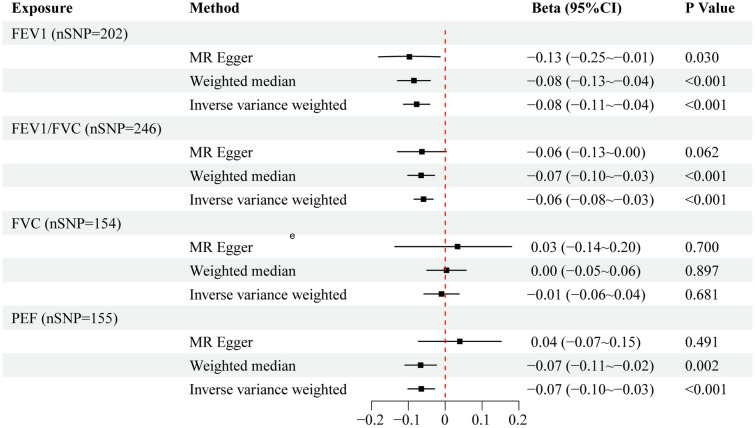
The forest plot shows the causal estimates between FEV1, FEV1/FVC, FVC, PEF, and frailty. Three statistical methods, including inverse variance weighted, MR-Egger, and weighted median, were used in calculating estimates. MR, Mendelian randomization; nSNP, number of single nucleotide polymorphism; FEV1, forced expiratory volume in the first second; FVC, forced vital capacity; PEF, peak expiratory flow

**Fig. 3:**
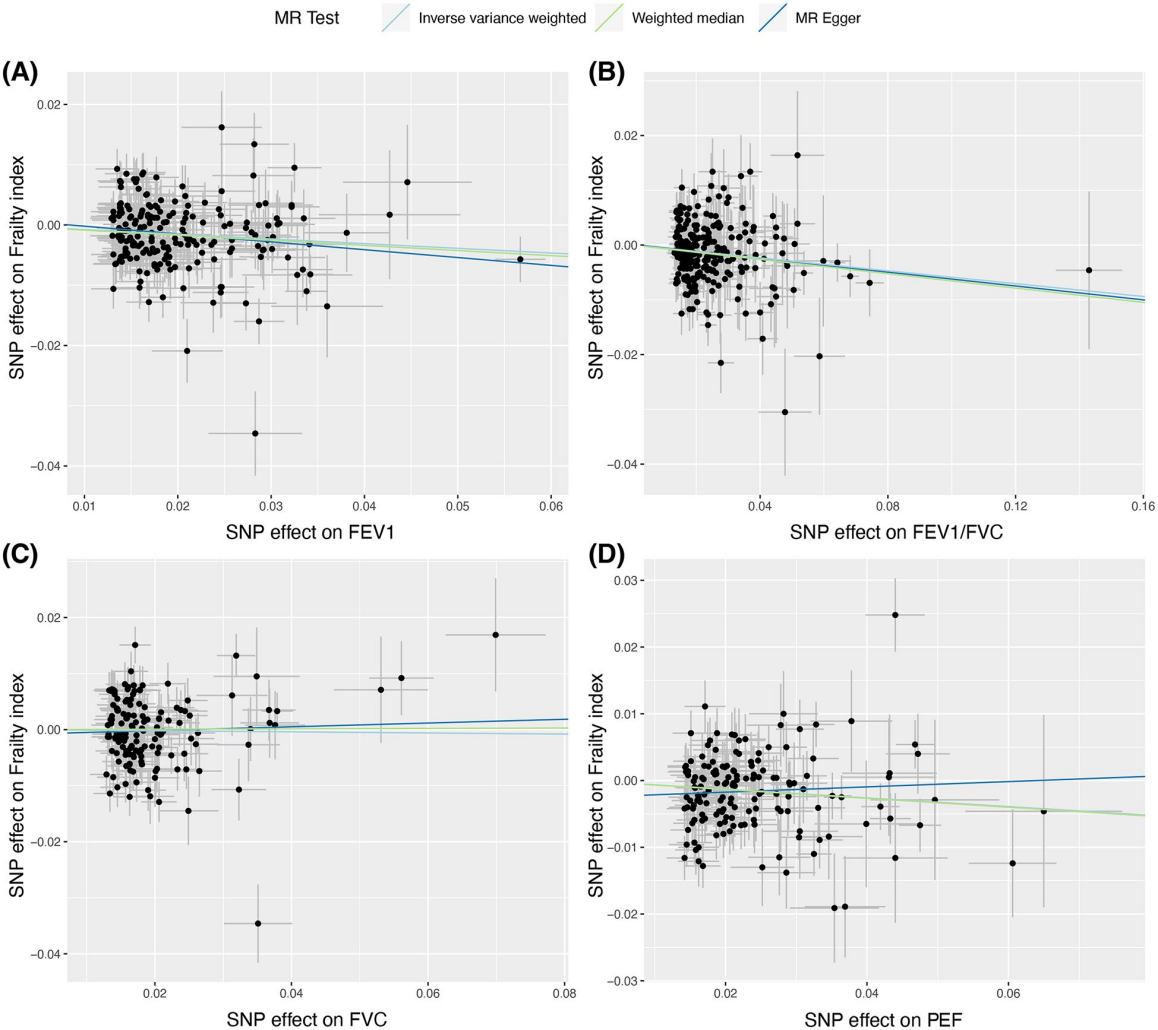
The scatter plot represents the causal effect of lung function on frailty in this MR study. Inverse variance weighted, weighted median, and MR-Egger approaches were employed to evaluate the causal association between lung function between frailty. **A** The causal linkage between FEV1 and frailty; **B** The causal linkage between FEV1/FVC and frailty; **C** The causal linkage between FVC and frailty; **D** The causal linkage between PEF and frailty. MR, Mendelian randomization; SNP, single nucleotide polymorphism; FEV1, forced expiratory volume in the first second; FVC, forced vital capacity; PEF, peak expiratory flow

There were 202, 246, 155, and 154 IV available for FEV1, FEV1/FVC, PEF, and FVC, respectively. All the IV had *F*-statistics larger than 10, indicating lack of weak instrumental bias in this MR study. Using the IVW method, genetically determined FEV1 was significantly associated with a decreased FI (*β* = − 0.08, 95% confidence interval [CI] = −0.11 to −0.04, *P* = 2.03E−05). Similarly, a significantly negative correlation was also found between FEV1/FVC and FI (*β* = − 0.06, 95% CI = − 0.08 to− 0.03, *P* = 9.51E−06). In addition, genetically predicted higher PEF was also linked to a lower FI (*β* = − 0.07, 95% CI = − 0.10 to − 0.03, *P* = 4.09E−04). However, there was no significant causal relationship between FVC and FI (*β* = – 0.01, 95% CI = − 0.06 to 0.04, *P* = 0.681) (Fig. 2). Sensitivity analyses using MR-Egger and weighted median approaches obtained estimates with the same direction as the results of the IVW approach, as demonstrated in the scatter plots (Fig. 3). The MR-Egger intercept test did not indicate the presence of horizontal pleiotropy between FEV1, FEV1 /FVC, PEF and FI (FEV1, *P* = 0.354; FEV1/FVC, *P* = 0.883; FVC, *P* = 0.602; PEF, *P* = 0.056). There was potential heterogeneity observed in this MR study and a random-effects IVW method was used (FEV1, *Q* = 370.72; FEV1/FVC, *Q* = 394.62; FVC, *Q* = 347.28; PEF, *Q* = 284.76) (Table 2). Although there were outliers founded in the MR-PRESSO test, the estimates between FEV1, FEV1/FVC, PEF and FI using MR-PRESSO corrected approach remained significant after removing the outliers (FEV1, *β* = −0.07, *P* = 7.70E−05; FEV1/FVC, *β* = −0.06, *P* = 9.24E−06; PEF, *β* = −0.07, *P* = 1.48E−04) (Table 2). The statistical power for FEV1, FEV1/FVC, FVC, and PEF was 100%, 100%, 9.4%, and 99.7% respectively, enhancing the reliability of the MR estimates. The results of leave-one-out sensitivity test demonstrated that excluding certain SNP did not have a fundamental impact on the overall estimates, indicating that the MR results were robust (Additional file 1: Figure S1).

**Table 2:** The MR-Egger intercept, MR-PRESSO, and heterogeneity tests for the causal relationship between lung function and frailty

Exposure	Method	Frailty (outcome)			
		Beta	*P*	Cochran *Q*	MR-Egger intercept (*P*)
FEV1	MR-PRESSO Raw	−0.08	3.11E−05	370.72	0.354
	MR-PRESSO Outlier-corrected	−0.07	7.70E−05		
FEV1/FVC	MR-PRESSO Raw	−0.06	1.43E−05	394.62	0.883
	MR-PRESSO Outlier-corrected	−0.06	9.24E−06		
FVC	MR-PRESSO Raw	−0.01	0.682	347.28	0.602
	MR-PRESSO Outlier-corrected	−0.00	0.865		
PEF	MR-PRESSO Raw	−0.07	5.40E−04	284.76	0.056
	MR-PRESSO Outlier-corrected	−0.07	1.48E−04		

*FEV1* forced expiratory volume in the first second, *FVC* forced vital capacity, *PEF* peak expiratory flow, *MR-PRESSO* MR-Pleiotropy RESidual Sum and Outlier, *N* number, *P* P-value

## Discussion

Although previous observational studies have reported a connection between lung function and frailty, establishing a causal link is challenging owing to the influence of confounding factors and reverse causation. The present MR study provides evidence supporting a significant causal association between lung function impairment and frailty risk. Estimates from IVW and other statistical approaches consistently showed an inverse association between FEV1, FEV1/FVC, PEF and frailty, but no causal linked was observed between FVC and frailty.

Frailty is a geriatric syndrome characterized by sarcopenia, a reduction in endurance and stress tolerance, as well as an increase in vulnerability and dependence [27]. Increasing studies have showed that lung diseases, such as COPD [5, 28], interstitial lung disease (ILD), and idiopathic pulmonary fibrosis (IPF), were associated with the risk of frailty [29–31]. For instance, it was reported that the prevalence of frailty in COPD patients was two-fold higher than non-COPD patients [12]. Meanwhile, lung diffusion capacity was also found to be an independent predictor of frailty in ILD and IPF patients [29, 30]. The above lung diseases are often accompanied by impaired lung function, which could contribute to a higher susceptibility of frailty. Indeed, a five-year follow-up study of elderly community-dwelling individuals revealed an inverse correlation between FEV1, FVC, and frailty, regardless of the presence of respiratory diseases, while every liter increase in FVC and FEV1 could reduce the incidence of frailty by 40% to 75% [11]. In addition, previous studies showed that frail and pre-frail elderly individuals had a lower PEF as compared to non-frail individuals [14, 32], and PEF ≤ 350 L/min (males) and ≤ 220 L/min (females) were also used to discriminate the presence of frailty in the elderly population [32]. Consistently, our MR study showed that lower FEV1, FEV1/FVC ratio, and PEF were causally linked to higher risk of frailty. In fact, previous studies have demonstrated that pulmonary rehabilitation via physical exercise could significantly improve physical endurance, prevent acute exacerbation of chronic lung disease, and ameliorate sarcopenia and frailty [33, 34]. Taken together, these findings highlight the potential benefits of interventions that focus on improving lung function to prevent or mitigate frailty in the elderly population.

There are several possible explanations for the inverse relationship between lung function and frailty. Individuals with worse lung function are likely to be less physically active, which might increase the risk of frailty [35, 36]. In addition, lung function impairment is often associated with excessive oxidative stress and systemic inflammation, such as elevated levels of interleukin-6 (IL-6), tumor necrosis factor-alpha (TNF-α), and C-reactive protein (CRP), which has been implicated in the pathogenesis of frailty [37, 38]. Moreover, healthy lung function indicates optimized tissue oxygenation, which is essential for preserving muscle mass and function and may lessen frailty [39, 40]. These factors may interact in complex ways to mediate the impact of lung function on frailty. Further studies are warranted to assess the mechanisms underlying this association, which may be beneficial for reducing the burden of frailty in the elderly population.

The reliability of MR results depends on the fulfillment of three core assumptions previously described [16]. To satisfy the relevance and exclusion assumption, we selected SNPs strongly (*P* ≤ 5E−08) associated with lung function parameters and not associated with frailty as IV, which was further filtered using F-statistic values [41]. The independent assumption in MR stipulates that the selected SNPs are not correlated with confounding factors linked to both lung function and frailty. Although SNPs associated with previously reported confounding factors [21, 42], such as smoking, alcohol consumption, body mass index, cancer, diabetes, coronary artery disease, inflammatory bowel disease, have been removed using the PhenoScanner V2 [19], we cannot completely rule out the influence of other undetermined potential confounding factors. This MR study exhibited robust statistical power for the causal effect of FEV1, FEV1/FVC, and PEF on frailty (> 99%), suggesting a good reliability of our findings. However, it is worthy to note that the statistical power for FVC was 9.4%, which might contribute to the false result between FVC and frailty in the present MR study and need to be further confirmed.

This study has several strengths. First, the F-statistic value for each IV was larger than 10, excluding the potential impact of weak instrumental variables. Second, the results from MR-Egger intercept, MR-PRESSO, and leave-one-out test indicate that MR estimates are robust and not be affected by any individual SNP. Finally, three of the four indicators for lung function (FEV1, FEV1/FV, and PEF) showed significant association with the risk of frailty, consistently supporting the impact of lung function on frailty risk. Despite the above strengths, there are still some limitations to be addressed here. First, although the estimates obtained using MR-PRESSO test remained significant after excluding potential outliers, there was discernible heterogeneity within this study and a random-effect IVW method was used. Second, the GWAS summary statistics for both lung functions and FI include partial samples from the UK Biobank, which may bias the MR estimates of this two-sample MR analysis. Further studies with independent samples are required to confirm our findings. Third, the MR study is predicated on three main assumptions, which are essential for its validity and interpretation [16]. Despite no horizontal pleiotropy observed in MR-egger intercept test, other undetected pleiotropy could not be excluded. Fourth, although we have removed SNPs associated with potential confounding factors reported in previous studies, we could not fully exclude the impact of other undefined confounders on the overall estimates. Lastly, since our research was based on the individuals of European ancestry, whether these associations exist in other population needs to be further explored.

## Conclusions

In summary, the present MR analysis showed that FEV1, FEV1/FVC, and PEF were negatively associated with FI, suggesting that these indicators might be used for the prediction and assessment of frailty in clinical practice. These findings demonstrate that improving lung function may reduce the risk of frailty and slow its progression. Since frailty and lung function may alter over the course of time, further longitudinal research is warranted to determine the direction in which causality runs.

### Supplementary Information

**Additional file 1: Figure S1.** The leave-one-out plots for the causal associations between lung function and frailty. **A** showed that removing any SNP does not affect the estimates between FEV1 and frailty; **B** showed that excluding any SNP does not affect the estimates between FEV1/FVC and frailty; **C** showed that removing any SNP does not affect the estimates between FVC and frailty; **D** showed that removing any SNP does not affect the estimates between PEF and frailty. SNP, single nucleotide polymorphism; FEV1, forced expiratory volume in the first second; FVC, forced vital capacity; PEF, peak expiratory flow.

**Additional file 2: Table S1.** Definition and evaluation criteria for frailty phenotype.

**Additional file 3: Table S2.** Specific variables and related calculations the frailty index.

**Additional file 4: Table S3.** Demographic features of participants in two studies on lung function and frailty.

## Data Availability

The GWAS summary statistics data used in this study are all publicly available in Open GWAS.

## Abbreviations

CI: Confidence interval
COPD: Chronic obstructive pulmonary disease
CRP: C-reactive protein
FEV1: Forced expiratory volume in the first second
FI: Frailty index
FVC: Forced vital capacity
GWAS: Genome-wide association study
IL-6: Interleukin-6
ILD: Interstitial lung disease
IPF: Idiopathic pulmonary fibrosis
IV: Instrumental variables
IVW: Inverse-variance weighted
LD: Linkage disequilibrium
MR: Mendelian randomization
MR-PRESSO: MR Pleiotropy RESidual Sum and Outlier
OR: Odds ratio
PEF: Peak expiratory flow
PF: Pulmonary function
SNPs: Single nucleotide polymorphisms
TNF-α: Tumour necrosis factor alpha
