# Supplementary material for: Lung function and the risk of frailty in the European population: a mendelian randomization study
Source: Eur J Med Res. 2024 Feb 1;29:95. doi: 10.1186/s40001-024-01685-y (PMC10832278; doi:10.1186/s40001-024-01685-y)
Supplement: Supplementary file 1 — Additional file 1: Figure S1. The leave-one-out plots for the causal associations between lung function and frailty. A showed that removing any SNP does not affect the estimates between FEV1 and frailty; B showed that excluding any SNP does not affect the estimates between FEV1/FVC and frailty; C showed that removing any SNP does not affect the estimates between FVC and frailty; D showed that removing any SNP does not affect the estimates between PEF and frailty. SNP, single nucleotide polymorphism; FEV1, forced expiratory volume in the first second; FVC, forced vital capacity; PEF, peak expiratory flow. [file 40001_2024_1685_MOESM1_ESM.docx]

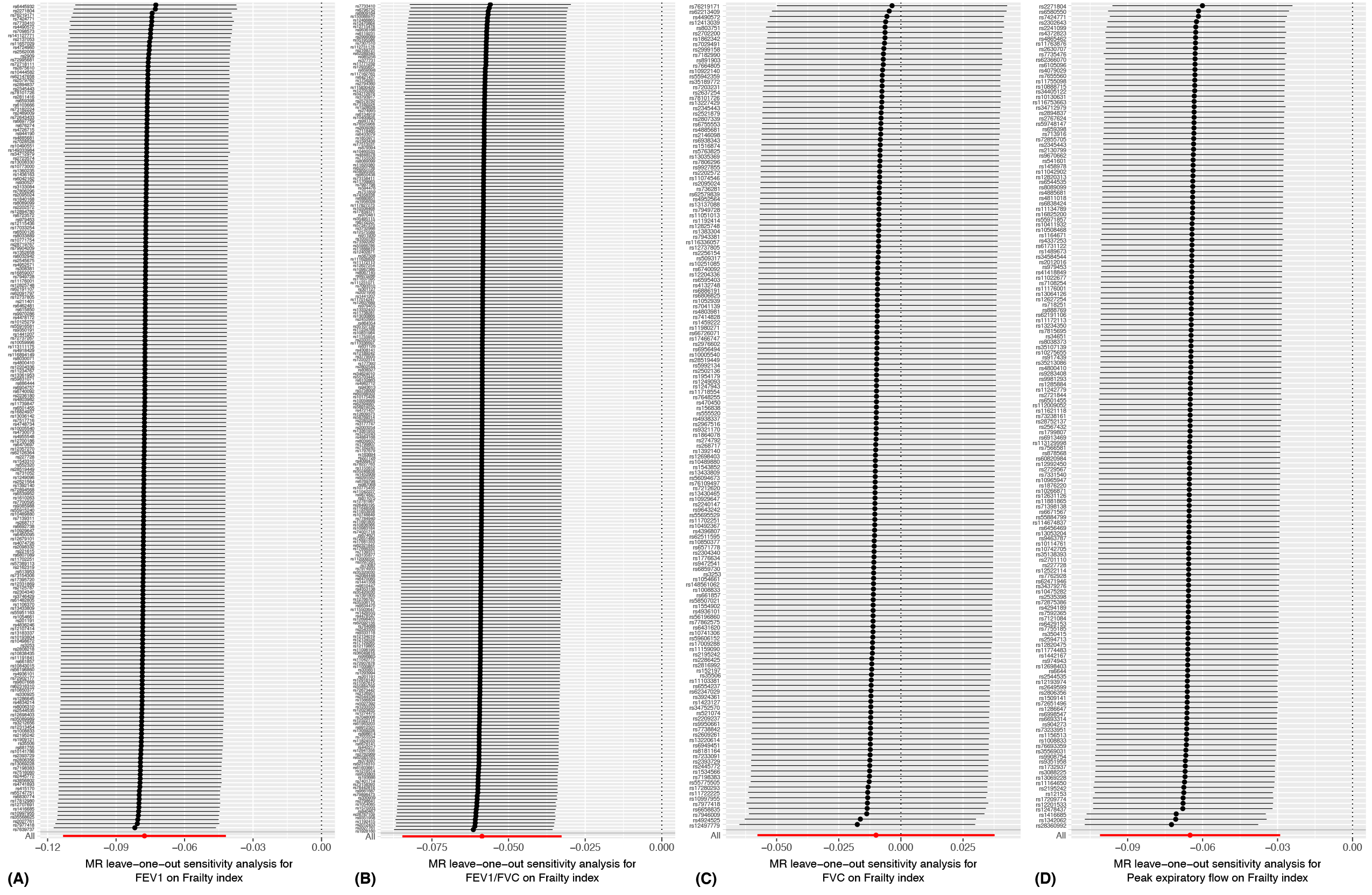


**Supplementary** **Figure S1.** The leave-one-out plots for the causal associations between lung function and frailty. **(A)** showed that removing any SNP does not affect the estimates between FEV1 and frailty; **(B)** showed that excluding any SNP does not affect the estimates between FEV1/FVC and frailty; **(C)** showed that removing any SNP does not affect the estimates between FVC and frailty; **(D)** showed that removing any SNP does not affect the estimates between PEF and frailty. SNP, single nucleotide polymorphism; FEV1, forced expiratory volume in the first second; FVC, forced vital capacity; PEF, peak expiratory flow.
