# Supplementary material for: Lung function and the risk of frailty in the European population: a mendelian randomization study
Source: Eur J Med Res. 2024 Feb 1;29:95. doi: 10.1186/s40001-024-01685-y (PMC10832278; doi:10.1186/s40001-024-01685-y)
Supplement: Supplementary file 2 — Additional file 2: Table S1. Definition and evaluation criteria for frailty phenotype. [file 40001_2024_1685_MOESM2_ESM.docx]

**Supplementary Table S1** Definition and evaluation criteria for frailty phenotype.

| **Frailty Phenotype** | |
| --- | --- |
| Unintentional weight loss |  |
| Self reported fatigue |  |
| Diminished physical activity |  |
| Measured impairment (comparative to  age-standardised norms) of grip strength |  |
| Gait speed |  |

*: Frailty phenotype is defined as three out of five variables outside the normative range.
