# Supplementary material for: Lung function and the risk of frailty in the European population: a mendelian randomization study
Source: Eur J Med Res. 2024 Feb 1;29:95. doi: 10.1186/s40001-024-01685-y (PMC10832278; doi:10.1186/s40001-024-01685-y)
Supplement: Supplementary file 3 — Additional file 3: Table S2. Specific variables and related calculations the frailty index. [file 40001_2024_1685_MOESM3_ESM.docx]

**Supplementary Table S2** Specific variables and related calculations the frailty index.

| **Frailty Index (FI)** | | | | | | | |
| --- | --- | --- | --- | --- | --- | --- | --- |
| **Symptoms** | **Past history** | **Signs** | **Physical exam** | **Neur exam** | **Disease classifications** | **Abnormal laboratory values** | **Others** |
| clouding/delirium | history of stroke | resting tremor | head and neck (normal, abnormal) | sucking, release sign | high blood pressure | Glucose | onset between ages 40 and 90 |
| sleep changes | headaches of recent onset | action tremor | thyroid | snout, release sign | heart and circulation problems | Potassium | release signs |
| mobility impairment | chronic visual loss | dyskinesias/chorea | breast | palmomentals R, release sign | stroke or effect of stroke | BUN | years since onset |
| difficulty with memory | difficulties with hearing | akinesia | lungs | tone/neck | eye trouble | Creatinine | ADL |
| difficulty with mood | arterial hypertension | impaired abstract thinking | cardiovascular | rone limb | ear trouble | Calcium | IADL |
| difficulty with going out | cardiac symptoms | impaired judgement | peripheral pulses | tremor/rest | chest problems | Phosp. |  |
| difficulty with cooking | respiratory complaints | aphasia | abdomen | tremor/action | lose control of bladder | TSH |  |
| difficulty with getting dressed | history of malignancy | apraxia | rectum | coordination/trunk | lose control of bowels | B12 |  |
| difficulty with grooming | gastro-intestinal complaints | agnosia | skin | coordination/limb | diabetes | Serum folate |  |
| difficulty with bath | urinary complaints |  |  | bradykinesia/limb | kidney trouble | VDRL |  |
| difficulty with toileting | history of thyroid disease |  |  | bradykinesia/face | Parkinsonís disease | Total protein |  |
| incontinence of urine | history of diabetes mellitus |  |  | posture/standing |  | Albumin |  |
| incontinence of stool |  |  |  | gait, motor system |  | Inorganic phosphate |  |
| onset of symptoms (gradual or abrupt) |  |  |  | vibration, sensory system |  | RBC folate |  |
| feel sad, blue or depressed |  |  |  | bulk |  | Sodium |  |
|  |  |  |  | diskinesia |  |  |  |

*: FI can be calculate by counting the dificits presented in the individual and dividing it by the total number of the dificits lists. For example, if an individual was given 92 tests and 23 from 92 variables were present, the frailty index is 23/92 = 0.25.

BUN, Blood urea nitrogen; TSH, Thyroid stimulating hormone; B12, Vitamin B12; VDRL, Venereal disease research laboratory test; RBC, Red Blood Cell Count; ADL, Activities of daily living; IADL, Instrumental Activity of Daily Living.
