# Supplementary material for: Lung function and the risk of frailty in the European population: a mendelian randomization study
Source: Eur J Med Res. 2024 Feb 1;29:95. doi: 10.1186/s40001-024-01685-y (PMC10832278; doi:10.1186/s40001-024-01685-y)
Supplement: Supplementary file 4 — Additional file 4: Table S3. Demographic features of participants in two studies on lung function and frailty. [file 40001_2024_1685_MOESM4_ESM.docx]

**Supplementary Table S3** Demographic features of participants in two studies on lung function and frailty.

|  | **Lung function** | **Frailty index (FI)** | |
| --- | --- | --- | --- |
|  |  | UK Blobank | TwinGene |
| **Sample** | 321,047 | 164,610 | 10,616 |
| **Populations** | European | European | Swedish |
| **Female, n (%)** | 178489 (55.6%) | 84819 (51.3%) | 5577 (52.5%) |
| **Age range (Y)** | 39-672 | 60-70 | 41-87 |
| **Mean age (Y, SD)** | 56.44 (8.02) | 64.1 (2.8) | 58.3 (7.9) |
| **Mean height (cm, SD)** | 168.57 (9.13) | NA | NA |
| **Frailty index, range** | NA | 0-27 | 0-26.25 |
